# Supplementary material for: A UMLS-based spell checker for natural language processing in vaccine safety
Source: BMC Med Inform Decis Mak. 2007 Feb 12;7:3. doi: 10.1186/1472-6947-7-3 (PMC1805499; doi:10.1186/1472-6947-7-3)
Supplement: Additional file 13 — RAP application directory. Sets up directory for RAP (RDF application for PHP) source code [file 1472-6947-7-3-S13.gz › rap/doc/phpdoc/elementindex_resModel.html]

xml version="1.0" encoding="iso-8859-1"?


## [resModel] element index

### Package indexes

- model
- dataset
- infModel
- ontModel
- syntax
- rdql
- utility

All elements
  

a
c
e
f
g
h
i
k
l
n
r
s
u
v
w

a

top

add
:   ResModel::add() in ResModel.php

    Adds a new triple to the Model without checking if the statement is already in the Model.

add
:   ResList::add() in ResList.php

    Add the given value to the end of the list.

add
:   ResContainer::add() in ResContainer.php

    Add a new value to a container.

addAtIndex
:   ResSeq::addAtIndex() in ResSeq.php

    Insert a new member into the sequence at the specified position.

addModel
:   ResModel::addModel() in ResModel.php

    Adds another model to this MemModel.

addNamespace
:   ResModel::addNamespace() in ResModel.php

    Adds a namespace and prefix to the model.

addParsedNamespaces
:   ResModel::addParsedNamespaces() in ResModel.php

    Adds the namespaces to the model. This method is called by the parser. !!!! addParsedNamespaces() not overwrites manual added namespaces in the model !!!!

addProperty
:   ResResource::addProperty() in ResResource.php

    Add a property to this resource.

addWithoutDuplicates
:   ResModel::addWithoutDuplicates() in ResModel.php

    Checks if a new statement is already in the Model and adds the statement, if it is not in the Model.

append
:   ResList::append() in ResList.php

    Answer a new list that is formed by adding each element of

c

top

concatenate
:   ResList::concatenate() in ResList.php

    Change the tail of this list to point to the given list, so that this list becomes the list of the concatenation of the elements of both lists. This is a side-effecting operation on this list; for a non side-effecting alternative, see append.

cons
:   ResList::cons() in ResList.php

    Return a reference to a new list cell whose head is value and whose tail is this list.

contains
:   ResList::contains() in ResList.php

    Answer true if the given node appears as the value of a value of any of the cells of this list.

contains
:   ResContainer::contains() in ResContainer.php

    Determine whether the container contains a value

contains
:   ResModel::contains() in ResModel.php

    Tests if the Model contains the given statement.

containsAll
:   ResModel::containsAll() in ResModel.php

    Determine if all of the statements in a model are also contained in this model.

containsAny
:   ResModel::containsAny() in ResModel.php

    Determine if any of the statements in a model are also contained in this model.

containsResource
:   ResModel::containsResource() in ResModel.php

    Determine if the node (ResResource / ResLiteral) $node appears in any statement of this model.

copy
:   ResList::copy() in ResList.php

    Answer a list that contains all of the elements of this list in the same order, but is a duplicate copy in the underlying model.

createAlt
:   ResModel::createAlt() in ResModel.php

    Create a new RDF Container from type rdf:Alt This method may return an existing container with the correct URI and model, or it may construct a fresh one, as it sees fit.

createBag
:   ResModel::createBag() in ResModel.php

    Create a new RDF Container from type rdf:Bag This method may return an existing container with the correct URI and model, or it may construct a fresh one, as it sees fit.

createList
:   ResModel::createList() in ResModel.php

    Create a new RDF Collection from type rdf:List This method may return an existing container with the correct URI and model, or it may construct a fresh one, as it sees fit.

createLiteral
:   ResModel::createLiteral() in ResModel.php

    Create an untyped literal from a String value with a specified language.

createProperty
:   ResModel::createProperty() in ResModel.php

    Create a new Property associated with this model.

createResource
:   ResModel::createResource() in ResModel.php

    Create a new resource associated with this model.

createSeq
:   ResModel::createSeq() in ResModel.php

    Create a new RDF Container from type rdf:Seq This method may return an existing container with the correct URI and model, or it may construct a fresh one, as it sees fit.

createTypedLiteral
:   ResModel::createTypedLiteral() in ResModel.php

    Create a literal from a String value with the $dtype Datatype An existing literal of the right value may be returned, or a fresh one created.

current
:   ResIterator::current() in ResIterator.php

    Returns the current item

e

top

equals
:   ResResource::equals() in ResResource.php

    Checks if the resource equals another resource.

equals
:   ResModel::equals() in ResModel.php

    Checks if two models are equal.

f

top

find
:   ResModel::find() in ResModel.php

    General method to search for triples.

findFirstMatchingStatement
:   ResModel::findFirstMatchingStatement() in ResModel.php

    Searches for triples and returns the first matching statement.

g

top

get
:   ResList::get() in ResList.php

    Returns the value of the list element at the specified position or null.

getAssociatedModel
:   ResResource::getAssociatedModel() in ResResource.php

    Get the reference to the assocoated model.

getAssociatedModel
:   ResLiteral::getAssociatedModel() in ResLiteral.php

    Get the reference to the assocoated model.

getBaseURI
:   ResModel::getBaseURI() in ResModel.php

    Return current baseURI.

getContentInArray
:   ResList::getContentInArray() in ResList.php

    Get all values in the list as an array of ResResources

getDefault
:   ResAlt::getDefault() in ResAlt.php

    Return the default value for this resource

getHead
:   ResList::getHead() in ResList.php

    Get the value that is associated with the head of the list.

getIsAnon
:   ResResource::getIsAnon() in ResResource.php

    Determine whether this resource is an anonymous resource

getMember
:   ResSeq::getMember() in ResSeq.php

    Get the member at a given index

getMembers
:   ResContainer::getMembers() in ResContainer.php

    Get an array of all resources that are values of this container

getModel
:   ResModel::getModel() in ResModel.php

    Returns a reference to the underlying model (Mem/DB/InfModel) that contains the statements

getParsedNamespaces
:   ResModel::getParsedNamespaces() in ResModel.php

    Returns the models namespaces.

getProperty
:   ResModel::getProperty() in ResModel.php

    Answer a statement find(s, p, null) with ResResources(ResLiterals) from this model.

getProperty
:   ResResource::getProperty() in ResResource.php

    Answer some statement (this, p, O) in the associated model.

getTail
:   ResList::getTail() in ResList.php

    Get the list that is the tail of this list.

h

top

hasProperty
:   ResResource::hasProperty() in ResResource.php

    Determine whether this resource has any values for a given property.

i

top

indexOf
:   ResList::indexOf() in ResList.php

    Get the Position of the first occurrence of the given value in the list, or -1 if the value is not in the list.

indexOf
:   ResSeq::indexOf() in ResSeq.php

    Return the index of a given member of the sequence.

intersect
:   ResModel::intersect() in ResModel.php

    Returns a new model containing all the statements which are in both this model and another.

isAlt
:   ResContainer::isAlt() in ResContainer.php

    Returns true, if this resource is a container from type rdf:alt

isBag
:   ResContainer::isBag() in ResContainer.php

    Returns true, if this resource is a container from type rdf:bag

isEmpty
:   ResModel::isEmpty() in ResModel.php

    Checks if MemModel is empty

isEmpty
:   ResList::isEmpty() in ResList.php

    Returns true, if this list is empty

isSeq
:   ResContainer::isSeq() in ResContainer.php

    Returns true, if this resource is a container from type rdf:seq

isValid
:   ResList::isValid() in ResList.php

    Answer true if the list is well-formed, by checking that each node is correctly typed, and has a head and tail pointer from the correct vocabulary

k

top

key
:   ResIterator::key() in ResIterator.php

    Returns the key of the current item

l

top

listObjects
:   ResModel::listObjects() in ResModel.php

    Returns a ResIterator with all objects in a model.

listObjectsOfProperty
:   ResModel::listObjectsOfProperty() in ResModel.php

    Returns a ResIterator with all objects with a given property and property value.

listProperties
:   ResResource::listProperties() in ResResource.php

    List all the values with the property p as statements in an array.

listSubjects
:   ResModel::listSubjects() in ResModel.php

    Returns a ResIterator with all subjects in a model.

listSubjectsWithProperty
:   ResModel::listSubjectsWithProperty() in ResModel.php

    Returns a ResIterator with all subjects with a given property and property value.

load
:   ResModel::load() in ResModel.php

    Load a model from a file containing RDF, N3 or N-Triples.

n

top

next
:   ResIterator::next() in ResIterator.php

    Moves Iterator to the next item in the list

r

top

rdqlQuery
:   ResModel::rdqlQuery() in ResModel.php

    Perform an RDQL query on this MemModel.

rdqlQueryAsIterator
:   ResModel::rdqlQueryAsIterator() in ResModel.php

    Perform an RDQL query on this MemModel.

remove
:   ResList::remove() in ResList.php

    Remove the given value from this list.

remove
:   ResModel::remove() in ResModel.php

    Removes the statement of ResResources(ResTriples) from the MemModel.

remove
:   ResContainer::remove() in ResContainer.php

    Remove a value from the container.

removeAll
:   ResResource::removeAll() in ResResource.php

    Delete all the statements with predicate p for this resource from its associated model.

removeAtIndex
:   ResSeq::removeAtIndex() in ResSeq.php

    Remove the member at the specified index.

removeHead
:   ResList::removeHead() in ResList.php

    Remove the head of the list. The tail of the list remains in the model. Note that no changes are made to list cells that point to this list cell as their tail.

removeList
:   ResList::removeList() in ResList.php

    Remove all of the components of this list from the model.

removeNamespace
:   ResModel::removeNamespace() in ResModel.php

    removes a single namespace from the model

removeProperties
:   ResResource::removeProperties() in ResResource.php

    Delete all the properties for this resource from the associated model.

replace
:   ResList::replace() in ResList.php

    Replace the value at the i'th position in the list with the given value

ResAlt
:   ResAlt::ResAlt() in ResAlt.php

    Constructor You can supply a URI

ResAlt
:   ResAlt in ResAlt.php

    This interface defines methods for accessing RDF Alternative resources.

ResBag
:   ResBag in ResBag.php

    This interface defines methods for accessing RDF Bag resources.

ResBag
:   ResBag::ResBag() in ResBag.php

    Constructor You can supply a URI

ResContainer
:   ResContainer in ResContainer.php

    An RDF Container.

ResContainer
:   ResContainer::ResContainer() in ResContainer.php

    Constructor You can supply a URI

ResIterator
:   ResIterator::ResIterator() in ResIterator.php

    Constructor.

ResIterator
:   ResIterator in ResIterator.php

    Implementation of a resource iterator.

ResList
:   ResList in ResList.php

    Implementation of an rdf:Collection (rdf:List) Provides a convenience encapsulation for lists formed from chains of RDF statements arranged to form a head/tail cons-cell structure.

ResList
:   ResList::ResList() in ResList.php

    Constructor You can supply a URI

ResLiteral
:   ResLiteral::ResLiteral() in ResLiteral.php

    Constructor You have to supply a string.

ResLiteral
:   ResLiteral in ResLiteral.php

    An RDF literal.

ResModel
:   ResModel::ResModel() in ResModel.php

    Constructor You have to supply a memmodel/dbmodel/infmodel to save the statements.

ResModel
:   ResModel in ResModel.php

    A ResModel provides an resource centric view on an underlying RDF model.

ResAlt.php
:   ResAlt.php in ResAlt.php

ResBag.php
:   ResBag.php in ResBag.php

ResContainer.php
:   ResContainer.php in ResContainer.php

ResIterator.php
:   ResIterator.php in ResIterator.php

ResList.php
:   ResList.php in ResList.php

ResLiteral.php
:   ResLiteral.php in ResLiteral.php

ResModel.php
:   ResModel.php in ResModel.php

ResProperty.php
:   ResProperty.php in ResProperty.php

ResResource.php
:   ResResource.php in ResResource.php

ResSeq.php
:   ResSeq.php in ResSeq.php

ResProperty
:   ResProperty in ResProperty.php

    An RDF Property.

ResProperty
:   ResProperty::ResProperty() in ResProperty.php

    Constructor You can supply a URI

ResResource
:   ResResource::ResResource() in ResResource.php

    Constructor You can supply a uri

ResResource
:   ResResource in ResResource.php

    An RDF Resource.

ResSeq
:   ResSeq::ResSeq() in ResSeq.php

    Constructor You can supply a URI

ResSeq
:   ResSeq in ResSeq.php

    This interface defines methods for accessing RDF Sequence resources.

rewind
:   ResIterator::rewind() in ResIterator.php

    Resets iterator list to start

s

top

sameListAs
:   ResList::sameListAs() in ResList.php

    Answer true if this list has the same elements in the same order as the given list. Note that the standard equals test just tests for equality of two given list cells.

saveAs
:   ResModel::saveAs() in ResModel.php

    Saves the RDF,N3 or N-Triple serialization of the MemModel to a file.

set
:   ResSeq::set() in ResSeq.php

    Set the value at a given index in the sequence.

setAssociatedModel
:   ResResource::setAssociatedModel() in ResResource.php

    Sets the reference to the assocoated model.

setAssociatedModel
:   ResLiteral::setAssociatedModel() in ResLiteral.php

    Sets the reference to the assocoated model.

setBaseURI
:   ResModel::setBaseURI() in ResModel.php

    Set a base URI for the MemModel.

setDefault
:   ResAlt::setDefault() in ResAlt.php

    Set the default value of this container.

setFindLiteralWithLang
:   ResIterator::setFindLiteralWithLang() in ResIterator.php

    Sets that only Literals with the matching language should be returned

setHead
:   ResList::setHead() in ResList.php

    Update the head of the list to have the given value, and return the previous value.

setIsAnon
:   ResResource::setIsAnon() in ResResource.php

    Set whether this resource is an anonymous resource

setTail
:   ResList::setTail() in ResList.php

    Update the list cell at the front of the list to have the given list as tail. The old tail is returned, and remains in the model.

setURI
:   ResResource::setURI() in ResResource.php

    Sets the URI of this resource

size
:   ResModel::size() in ResModel.php

    Number of statements in the MemModel

size
:   ResContainer::size() in ResContainer.php

    Returns the number values in the container.

subtract
:   ResModel::subtract() in ResModel.php

    Returns a new model that is the subtraction of another model from this model.

u

top

unite
:   ResModel::unite() in ResModel.php

    Returns a new Model that is the set-union of the model with another model.

v

top

valid
:   ResIterator::valid() in ResIterator.php

    Says if there are additional items left in the list

w

top

writeAsHTMLTable
:   ResModel::writeAsHTMLTable() in ResModel.php

    Writes the RDF serialization of the MemModel as HTML table.

writeRdfToString
:   ResModel::writeRdfToString() in ResModel.php

    Writes the RDF serialization of the MemModel as HTML table.

a
c
e
f
g
h
i
k
l
n
r
s
u
v
w
